# Evaluation of coronary artery disease in patients with atrial fibrillation by cardiac computed tomography for catheter ablation: CADAF-CT trial

**DOI:** 10.1007/s00380-020-01572-6

**Published:** 2020-03-05

**Authors:** Takahiro Mito, Masao Takemoto, Yoshibumi Antoku, Akihiro Masumoto, Masatsugu Nozoe, Satoko Kinoshita, Atsushi Tanaka, Yusuke Yamamoto, Takafumi Ueno, Takuya Tsuchihashi

**Affiliations:** 1 Cardiology, Munakata Suikokai General Hospital, Fukutsu, Japan; 2 Cardiovascular Center, Steel Memorial Yawata Hospital, 1-1-1 Haruno-machi, Yahatahigashi-ku, Kitakyushu, 805-8508 Japan; 3 Cardiology, Fukuoka Kinen Hospital, Fukuoka, Japan; 4 Cardiology, Saiseikai Fukuoka General Hospital, Fukuoka, Japan

**Keywords:** Atrial fibrillation, Catheter ablation, CHADS_2_ score, Computed tomography, Coronary artery disease, Myocardial ischemia

## Abstract

Almost all institutions routinely perform cardiac computed tomography (CT) before radiofrequency catheter ablation (RFCA) of atrial fibrillation (AF) to evaluate the cardiac anatomy. The ideal timing of the CT image acquisition is different between for RFCA of AF and for evaluation of coronary artery lesions (CALs). Thus, the aim of this study was to assess whether 64- or 320-line routine cardiac CT scans before RFCA of AF could evaluate both coronary artery lesions and pulmonary veins (LA-PVs) anatomy at the timing of the image acquisition of the LA-PVs in patients with AF who underwent RFCA of AF. The CALs were evaluated in 606 consecutive patients who underwent RFCA of AF assessed by the ideal timing of the CT image acquisition for RFCA of AF, and myocardial ischemia (MI) was also evaluated in patients with severe coronary stenosis (≥ 50%) and unevaluable CALs due to their severe coronary calcification and banding artifact by additional examinations combined with exercise stress testing, ^201^Tl scintigraphy, and/or fractionated flow reserve measurements. This study revealed that, in patients with AF who underwent RFCA of AF, (1) both 64- and 320-line cardiac CT scans for RFCA of AF could evaluate CALs in 93% of those patients, (2) the prevalence of MI was 9%, (3) significant relationships between the CHADS_2_ score and prevalence of MI were observed (*p* = 0.003), and (4) the positive predict values of MI in patients with severe coronary stenosis (≥ 50%) and unevaluable CALs also significantly increased in accordance with the CHADS_2_ score (*p* = 0.003). The evaluation of CALs and MI by routine cardiac CT for RFCA of AF combined with the additional examinations may be one of the most feasible modalities for patients with AF.

## Introduction

The number of patients with atrial fibrillation (AF) has been increasing, and radiofrequency catheter ablation (RFCA) of AF has proven to be a useful strategy worldwide [1]. The CHADS_2_ score is well known to be a useful predictor of the risk for not only cerebral infarctions associated with AF [2], but also a new onset of AF [3]. Moreover, the CHADS_2_ score is constructed by risk factors of coronary artery disease (CAD), and has also been reported as a predictor of cardiovascular/cerebrovascular events in patients with CAD without AF [4, 5]. The close relationship between AF and CAD has been reported [6], and that (1) the prevalence of CAD detected by coronary computed tomography (CT) is significantly higher in patients with AF than in those without [7], and (2) coronary angiography (CAG) can detect coronary narrowing (≥ 50%) in patients with AF who undergo RFCA of AF [8]. Almost all institutions routinely perform cardiac CT before RFCA of AF to evaluate the cardiac anatomy, especially of the left atrium and pulmonary veins (LA-PVs). The ideal timing of the CT image acquisition is different between the LA-PVs and coronary arteries. For example, the imagings which were the ideal timings of the image acquisition of LA-PVs or coronary arteries starts after confirming the pulmonary arteries or ascending aorta have been contrasted by visual observation, respectively. Thus, the aim of this study was to assess whether 64- or 320-line routine cardiac CT scans before RFCA of AF could evaluate both coronary artery lesions and LA-PVs anatomy at the timing of the image acquisition of LA-PVs in patients with AF who underwent RFCA of AF.

## Materials and methods

### Study population and laboratory analysis

This study was approved by the institutional review committee and ethics review board of our hospitals. From April 2016 to June 2018, 606 consecutive patients (402 males and 204 females with a mean age of 69 ± 0.4 years and body surface area of 1.68 ± 0.01 m^2^) (Table 1) with AF and without previous histories of CAD who were admitted to our hospitals to undergo RFCA of AF were evaluated. The type of AF was determined according to the 2011 ACC/AHA/ESC guidelines for the management of patients with AF [9]. Patients that could not use contrast because of renal dysfunction (serum creatinine ≥ 1.5 mg/dL) and that underwent hemodialysis were excluded. All patients had their history recorded and underwent a physical examination and laboratory analysis.

**Table 1:** Patient characteristics

	All	64-line	320-line	*p* value
*n*	606	410 (68%)	196 (32%)	–
Male	408 (67%)	268 (65%)	140 (71%)	0.137
Age (years)	69 ± 9.0	69 ± 9.2	68 ± 8.4	0.075
Body mass index (kg/m^2^)	23.1 ± 3.5	23.3 ± 3.5	22.8 ± 3.6	0.146
Body surface area (m^2^)	1.68 ± 0.19	1.67 ± 0.19	1.69 ± 0.17	0.082
CHADS_2_ score	2.05 ± 1.29	2.04 ± 1.30	2.07 ± 1.29	0.791
0	56 (9%)	38 (9%)	18 (9%)	0.981
1	163 (27%)	110 (27%)	53 (27%)	0.941
2	200 (33%)	140 (34%)	60 (31%)	0.332
3	108 (18%)	70 (17%)	38 (19%)	0.432
4	47 (8%)	32 (8%)	15 (8%)	0.955
≥ 5	32 (5%)	20 (5%)	12 (6%)	0.430
Congestive heart failure	293 (48%)	188 (46%)	105 (54%)	0.243
Hypertension	424 (70%)	287 (70%)	137 (70%)	0.899
Age (≥ 75 years old)	164 (27%)	122 (33%)	42 (21%)	0.053
Diabetes mellitus	158 (26%)	107 (26%)	51 (26%)	0.941
History of CVA/TIA	103 (17%)	66 (16%)	37 (19%)	0.255
Dyslipidemia	200 (33%)	135 (33%)	65 (33%)	0.951
Ex or current smoking	170 (28%)	115 (28%)	55 (28%)	0.977
Type of atrial fibrillation				
Paroxysmal	369 (61%)	240 (59%)	129 (66%)	0.086
Persistent	209 (33%)	146 (36%)	62 (32%)	0.336
Long-lasting	29 (5%)	24 (6%)	5 (3%)	0.075
Laboratory analysis				
Serum creatinine (mg/dl)	0.89 ± 0.24	0.89 ± 0.24	0.90 ± 0.23	0.409
Left-ventricular ejection fraction (%)	63 ± 9.9	63 ± 9.1	64 ± 11	0.268
Diameter of left atrium (mm)	39 ± 6.7	39 ± 6.6	39 ± 6.7	0.309
Parameters during CT imaging				
Heart rate (bpm)	66 ± 15	66 ± 15	66 ± 16	0.829
Sinus rhythm	398 (66%)	261 (64%)	137 (70%)	0.131
Medications during CT imaging				
Beta-blocker (%)	421 (69%)	293 (71%)	128 (65%)	0.124
Minor tranquilizer (%)	172 (28%)	119 (29%)	52 (27%)	0.524

*CVA *cerebrovascular apoplexy, *TIA* transient ischemic attack, *CT* computed tomography

### Computed tomography

All patients gave written informed consent before imaging. The imaging technique has been described previously [10]. In brief, after sublingual administration of 0.3 mg of nitroglycerin, following a test bolus, 50–60 ml of nonionic contrast medium (Omnipaque 300, Amersham Health, Oslo, Norway) was injected at 20–22 mgI/kg/s via an antecubital vein. The imaging which was the ideal timing of the image acquisition of LA-PVs started after confirming the pulmonary arteries had been contrasted by visual observation. Scanning was performed in a single breath hold in the cranio-caudal direction at the level of the aortic arch using a simultaneous acquisition of eight sections (each 0.5 mm), with a prospective electrocardiogram (ECG)-triggering set at 75% of the RR interval, beam collimation of 10 mm, and table speed 16.75 mm/0.5 s or fixed table (0.5 or 0.275 s tube rotation time, 120 kV, 500 mA, or 750 mA) by 64-line or 320-line CT scans. Contiguous 0.5 mm axial CT (Aquilion, 64-line, 320-line, TOSHIBA, Tokyo, Japan) slices were reconstructed from the CT data using a soft-tissue algorithm and the resulting DICOM data were recorded onto a CD-ROM. When the patients’ heart rate was more than 70 bpm, the oral administration of 20 mg of the beta-blocker metoprolol tartrate and/or 0.5 mg of the minor tranquilizer etizolam were used.

### Evaluation of coronary artery lesions by cardiac CT and the detection of myocardial ischemia

The coronary lesions were evaluated in all patients who underwent cardiac CT scans at the timing of the image acquisition of LA-PVs for RFCA of AF. Mild-to-moderate and severe stenoses were defined as ≤ 50% and > 50% stenosis, respectively. Unevaluable coronary artery lesions were defined as severe coronary calcifications and/or banding artifacts [11] that made the evaluation of the coronary artery lesions impossible. The patients with severe coronary stenosis (> 50%) and/or unevaluable coronary artery lesions were evaluated for myocardial ischemia before or after the RFCA of AF. To evaluate the myocardial ischemia, they underwent examinations combined with exercise stress testing, ^201^Tl scintigraphy, and/or fractionated flow reserve (FFR) measurements [12].

### Statistical analysis

The numerical results are expressed in the text as the mean ± standard deviation. Paired data were compared by a Fisher’s exact test and Student’s *t* test. The trend in the proportions and correlation between the prevalence of severe coronary stenosis (> 50%), unevaluable coronary artery lesions, or myocardial ischemia and the CHADS_2_ score was determined by a Cochran–Armitage analysis. All analyses were performed with SAS version 9.2 software (SAS Institute, Cary, NC, USA). A *p* of < 0.05 was considered to indicate statistical significance.

## Results

### Patient characteristics and laboratory analysis (Table 1)

Cardiac CT scans for RFCA of AF were performed in 606 patients with AF and without a previous history of CVD at our hospitals. Because three patients did not agree to the use of contrast for the cardiac CT because of their renal dysfunction, they were excluded from the statistical analysis. No patients suffered from any cardiac CT-related complications except for a contrast-induced eruption. In all patients, the mean CHADS_2_ score was 2.05 ± 1.29 points. The numbers of patients were 56 (9%), 163 (27%), 200 (33%), 108 (18%), 47 (8%), and 32 (5%) for those with a CHADS_2_ score of 0, 1, 2, 3, 4, and ≥ 5 point(s), respectively. The prevalence of congestive heart failure, hypertension, age over 75 year old, diabetes mellitus, history of cerebrovascular apoplexy/transient ischemic attack, dyslipidemia, ex- or current smoking, paroxysmal AF, persistent AF, and long-lasting AF was 239 (48%), 424 (70%), 164 (27%), 158 (26%), 103 (17%), 200 (33%), 170 (28%), 369 (61%), 209 (33%), and 29 (5%), respectively. The values of the serum creatinine, left-ventricular ejection fraction (LVEF), and diameter of the left atrium (LA) (LAD) by echocardiography were 0.89 ± 0.24 mg/dL, 63 ± 9.9%, and 39 ± 6.7 mm, respectively. The mean heart rare and prevalence of sinus rhythm during the CT imaging were 66 ± 15 bpm and 398 (66%), respectively. The prevalence of the use of beta-blockers and/or minor tranquilizers during the CT imaging was 421 (69%) and 172 (28%), respectively.

### Evaluation of the coronary artery lesions and myocardial ischemia (Tables 2, 3)

**Table 2:** Evaluation of coronary artery lesions

	All	64-line	320-line	*p* value
Number of patients	606	410 (68%)	196 (32%)	–
Coronary arterial stenosis				
Evaluable coronary artery lesions	562 (93%)	381 (93%)	181 (92%)	0.809
Mild-to-moderate (≤ 50%)	418 (69%)	283 (69%)	135 (69%)	0.801
Severe (> 50%)	144 (24%)	98 (24%)	46 (23%)	0.818
Unevaluable coronary artery lesions				
Severe coronary calcification	41 (7%)	26 (6%)	15 (8%)	0.548
Banding artifacts	3 (0.5%)	3 (0.7%)	0 (0%)	0.231

**Table 3:** Evaluation and detection of myocardial ischemia

Evaluation of myocardial ischemia	
Number of patients	188 (31%)
Exercise stress testing	180 (30%)
^ 201^Tl scintigraphy	82 (14%)
Fractionated flow reserve	62 (10%)
Detection of myocardial ischemia	
Number of patients	54 (9%)

The amount of the contrast medium and radiation exposure time when evaluating the LA-PV anatomy plus the coronary arteries were the almost same when compared with evaluating the LA-PV anatomy only. They were about 60 ml or 50 ml, and 20 s or 8 s by 64-line or 320-line CT scans, respectively. However, the extra time about 15 min was needed to reconstruction and evaluation of LA-PV anatomy plus the coronary arteries compared to LA-PV anatomy only. The coronary artery lesions in 562 (93%) out of the 606 patients could be evaluated, but not in the remaining 44 (7%) patients, defined as unevaluable coronary artery lesions, because of severe coronary calcifications (*n* = 41; 7%) or banding artifact (*n* = 3; 0.5%). The prevalence of mild-to-moderate (≤ 50%), severe stenosis (> 50%), and unevaluable coronary artery lesions, was 418 (69%), 144 (24%), and 44 (7%), respectively. Myocardial ischemia was evaluated in 188 (31%) patients, including 144 (24%) patients with a > 50% coronary artery stenosis and 44 (7%) with unevaluable coronary artery lesions by cardiac CT, exercise stress testing (30%), ^201^Tl scintigraphy (14%), and/or fractionated flow reserve (FFR) measurements [12] (10%). Finally, myocardial ischemia was detected in 54 (9%) patients. There were no patients, including both those 144 patients with severe (> 50%) coronary artery stenosis and the remaining 462 patients, who had major adverse cardiovascular events including acute coronary syndrome and heart failure before, during, and after RFCA of AF. After RFCA of AF, 51 patients out of 54 patients with myocardial ischemia underwent re-vascularization of coronary arteries with percutaneous coronary interventions. Remaining 3 patients with myocardial ischemia were treated by optimal medical therapies.

### Correlation between the CHADS_2_ score and myocardial ischemia (Fig. 1)

**Fig. 1:**
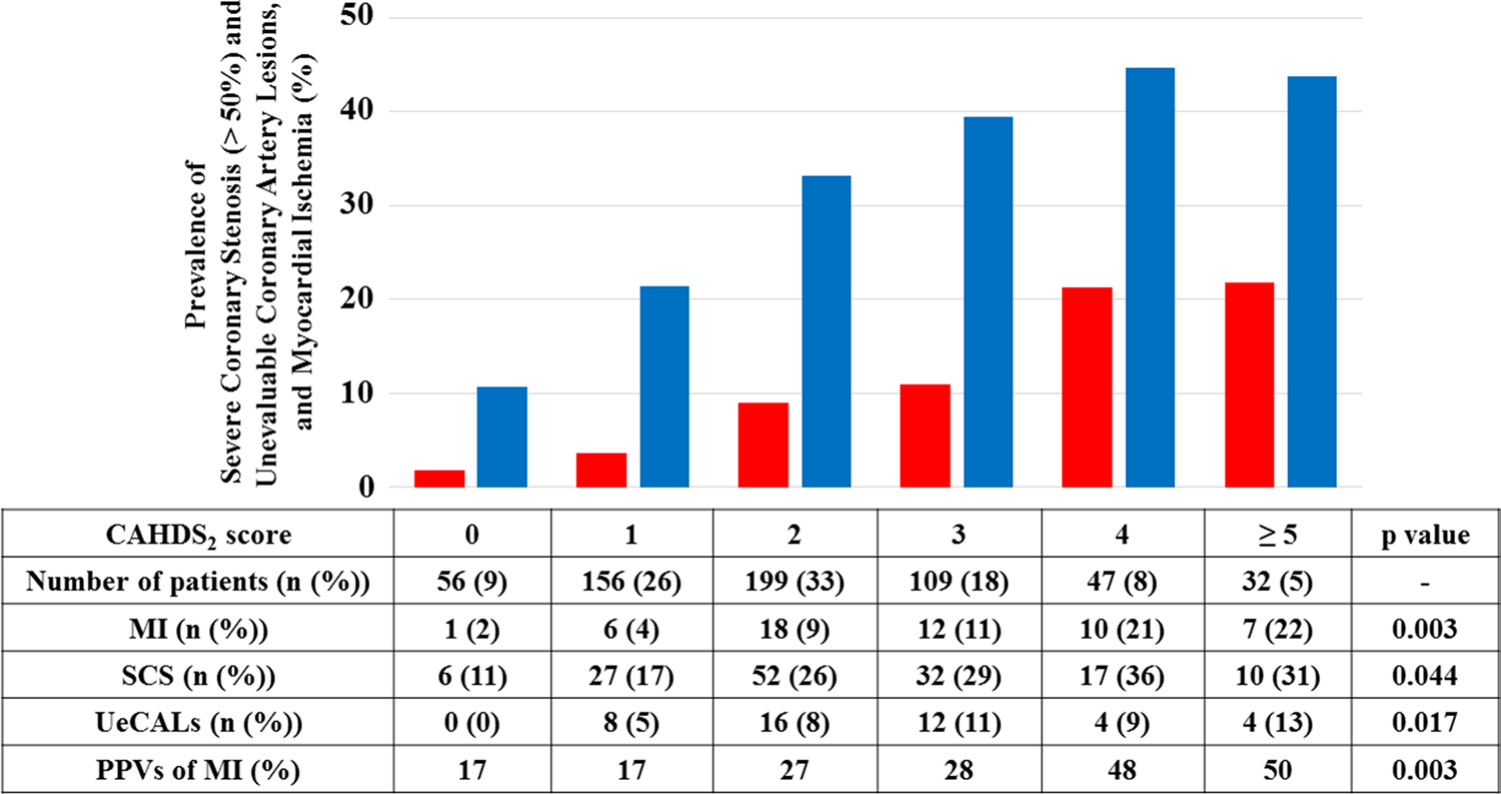
Prevalence of patients with severe coronary stenosis (SCS) (> 50%) and unevaluable coronary artery lesions (UeCALs) (blue bar), and myocardial ischemia (MI) (red bar) according to the CHADS_2_ score. In accordance with the CHADS_2_ score, the prevalence of those parameters (blue bar; *p* = 0.033 and red bar; *p* = 0.003) significantly increases. Moreover, in accordance with the CHADS_2_ score, the positive predict values (PPVs) of MI in patients with SCS and UeCALs significantly increase (*p* = 0.003). The numbers of patients were 56, 156, 199, 109, 47, and 32 for CHADS_2_ scores of 0, 1, 2, 3, 4, and ≥ 5 point(s), respectively

In accordance with the CHADS_2_ score, the prevalence of patients with severe coronary stenosis (> 50%) (*p* = 0.044) and unevaluable coronary artery lesions (blue bar) (*p* = 0.017), and myocardial ischemia (red bar) (*p* = 0.003) has been significantly increasing. Interestingly, in accordance with the CHADS_2_ score, the positive predict values of myocardial ischemia in patients with severe coronary stenosis (> 50%) and unevaluable coronary artery lesions also have been significantly increasing (*p* = 0.003).

## Comparison between the 64- and 320-line CT (Tables 1, 2)

The prevalence of the use of a 64- and 320-line CT scans was 410 (68%) and 196 (32%), respectively. There were no statistical differences in the patient characteristics (Table 1), prevalence of evaluable or unevaluable coronary artery lesions, and prevalence of mild, moderate, and severe coronary artery lesions (Table 2), between the two groups. However, there were only 3 (0.5%) patients with rapid AF (the mean heart rate: 91 ± 3 bpm) during the CT imaging whose coronary artery lesions could not be evaluated by the 64-line scans because of banding artifact [11] due to rapid AF, even though they received beta-blockers and minor tranquilizers (Table 1, 2).

## Discussion

This study revealed that, in patients with AF who underwent RFCA of AF, (1) both 64- and 320-line routine cardiac CT scans for RFCA of AF at the timing of the image acquisition of LA-PVs could evaluate coronary artery lesions in 93% of those patients, (2) the prevalence of myocardial ischemia was 9%, (3) significant relationships between the CHADS_2_ score and prevalence of severe coronary stenosis (> 50%) and unevaluable coronary artery lesions, and myocardial ischemia were observed, and (4) the positive predict values of myocardial ischemia in patients with severe coronary stenosis (> 50%) and unevaluable coronary artery lesions also significantly increased in accordance with the CHADS_2_ score (*p* = 0.003).

### Evaluation of the coronary artery lesions by cardiac CT scans for RFCA of AF

Because the scanning performed during a single breath hold with the prospective ECG-triggering set at 75% of the RR interval during the cardiac CT imaging, the scanning becomes very difficult during an AF rhythm that has an irregular RR interval and/or tachycardia of more than 100 bpm that has a shorter RR interval [13]. Those conditions sometimes cause banding artifact, which makes it difficult to evaluate coronary artery lesions. In addition, the unique problems of the 64-line cardiac CT include the approximately 20 s single breath hold. Because the approximately 20 s breath hold may be difficult for patients with lung disease and/or older patients such as those more than 80 years old, the 320-line cardiac CT scan, which has a shorter single breath hold time than the 64-line CT scan, may be more suitable for those older patients.

### Detection of myocardial ischemia by cardiac CT scans for RFCA of AF

Although it has previously been reported that the relatively high prevalence of CAD in patients with AF who undergo RFCA of AF and its relation to CHADS_2_ score have already been reported using CAG [8], CAG may be more invasive than the cardiac CT. This study revealed that the cardiac CT for RFCA of AF at the timing of the image acquisition of LA-PVs could evaluate coronary artery lesions in 93% of the patients who underwent RFCA of AF. Finally, myocardial ischemia was detected in 9% of those patients by cardiac CT for RFCA of AF at the timing of the image acquisition of LA-PVs combined with the additional examinations including with exercise stress testing, ^201^Tl scintigraphy, and/or fractionated flow reserve (FFR) measurements [12]. Moreover, a significant relationship between the CHADS_2_ score and myocardial ischemia (red bar in Fig. 1) (*p* = 0.003) was observed in this study. Furthermore, the positive predict value of myocardial ischemia in patients with severe coronary stenosis (> 50%) and unevaluable coronary artery lesions also significantly increased in accordance with the CHADS_2_ score (Fig. 1) (*p* = 0.003). These findings may support the previous findings that a high CHADS_2_ score has been proven to be a predictor of cardiovascular/cerebrovascular events in patients with documented CAD [4, 5]. Moreover, it has been reported that approximately 75% of culprit coronary artery lesions in patients who suffer from cardiovascular events such as acute coronary syndrome result from plaque ruptures developing a clot formation with mild-to-moderate coronary stenosis [14, 15]. Because 24% of the patients with AF who underwent RFCA of AF had severe coronary stenosis (> 50%) in this study, the future cardiovascular event risk may be decreased by performing primary prevention using diet, exercise, optimal medical therapies including statins, and re-vascularization in those patients.

### Positive predict values of myocardial ischemia

The positive predict values of myocardial ischemia in patients with severe coronary stenosis (> 50%) and unevaluable coronary artery lesions significantly increased in accordance with the CHADS_2_ score (Fig. 1) (*p* = 0.003). However, those values, especially in patients with less than 1 point with the CHADS_2_ score, were relatively low (17%) compared with more than 4 points with that score (about 50%). Moreover, it costs medical expenses for the additional examinations to evaluate myocardial ischemia. Thus, it is unknown whether it meets a law of nature to perform those examinations to evaluate myocardial ischemia in patients with severe coronary stenosis (> 50%) and unevaluable coronary artery lesions, even though their CHADS_2_ score is low.

### Limitations of the study

Although our study was a multi-center trial, it was limited by the retrospective design and its relatively small number of patients. Patients with AF who did not undergo RFCA of AF, especially with long-lasting AF, were not included in this study. Thus, whether our results can safely be extrapolated to a larger number of patients including patients with AF who do not undergo RFCA of AF should be determined in further prospective studies.

## Conclusions

This study revealed that both 64- and 320-line routine cardiac CT scans for RFCA of AF at the timing of the image acquisition of LA-PVs could be steadily detected coronary artery lesions. Moreover, the additional examinations combined with exercise stress testing, ^201^Tl scintigraphy, and/or fractionated flow reserve (FFR) measurements [12] could precisely detect myocardial ischemia in 9% of patients with AF who underwent RFCA of AF. Thus, physicians should be aware of this condition when examining patients with AF, especially in the presence of a high CHADS_2_ score, and that may be one of the most important risk factors for the progression of cardiovascular events in patients with AF. Finally, the evaluation of coronary artery lesions and myocardial ischemia by routine cardiac CT for RFCA of AF at the timing of the image acquisition of LA-PVs combined with the additional examinations including with exercise stress testing, ^201^Tl scintigraphy, and/or fractionated flow reserve (FFR) measurements [12] may be one of the most feasible modalities for patients with AF.

## Abbreviations

AF: Atrial fibrillation
BMI: Body mass index
CAG: Coronary angiography
CAD: Coronary artery disease
CT: Computed tomography
FFR: Fractionated flow reserve
RFCA: Radiofrequency catheter ablation
